# Identification of microRNAs in response to aluminum stress in the roots of Tibetan wild barley and cultivated barley

**DOI:** 10.1186/s12864-018-4953-x

**Published:** 2018-07-31

**Authors:** Liyuan Wu, Jiahua Yu, Qiufang Shen, Lu Huang, Dezhi Wu, Guoping Zhang

**Affiliations:** Department of Agronomy, College of Agriculture and Biotechnology, Zhejiang University, Hangzhou, 310058 China

**Keywords:** Tibetan wild barley, Aluminum resistance, miRNA, Small RNA sequencing, Root growth, Target genes

## Abstract

**Background:**

Barley is relatively sensitive to Aluminum (Al) toxicity among cereal crops, but shows a wide genotypic difference in Al tolerance. The well-known Al-tolerant mechanism in barley is related to Al exclusion mediated by a citrate transporter HvAACT1 (Al-activated citrate transporter 1). A 1-kb insertion in the promoter region of *HvAACT1* gene results in a dramatic increase of its expression level, which only occurs in some Al-tolerant cultivars. However, Al-tolerant Tibetan wild barley accession XZ29 did not have the 1-kb insertion.

**Results:**

We confirmed that the expression of *HvAACT1* and secretion of citrate and other organic acids did not explain the difference in Al-tolerant wild barley XZ29 and Al-sensitive cultivated barley Golden Promise. To identify microRNAs (miRNAs) and their target genes responsive to Al stress in barley roots, eight small RNA libraries with two biological replicates from these two genotypes exposed to control and Al-treated conditions were constructed and submitted to deep sequencing. A total of 342 miRNAs were identified in Golden Promise and XZ29, with 296 miRNAs being commonly shared in the two genotypes. Target genes of these miRNAs were obtained through bioinformatics prediction or degradome identification. Comparative analysis detected 50 miRNAs responsive to Al stress, and some of them were found to be exclusively expressed in XZ29 and associated with Al tolerance.

**Conclusions:**

miRNAs exclusively expressing in the wild barley were identified and found to be associated with Al stress tolerance. The current results provide a model of describing the roles of some special miRNAs associated with Al tolerance in the Tibetan wild barley.

**Electronic supplementary material:**

The online version of this article (10.1186/s12864-018-4953-x) contains supplementary material, which is available to authorized users.

## Background

Aluminum (Al) is a most abundant metal in the Earth’s crust. Although Al is nontoxic in the form of aluminosilicate minerals, toxicity arises when Al ions (Al^3+^) are solubilized from minerals and released into soils with the pH values below 5 [1]. It has been estimated that as much as approximately 50% of potentially arable lands in the world are acidic [2]. Al ions severely inhibit root growth and reduce the uptake of water and nutrients, thus finally reducing crop yields in acid soils [3].

To detoxify Al, some Al-tolerant plants have developed a variety of external and internal Al tolerant mechanisms. The external Al exclusion strategy is achieved by release of organic acids anions, such as citrate, malate and oxalate, from roots into rhizosphere to chelate Al [4–6]. Genes responsible for Al-induced citrate and malate release have been identified, which are members of *MATE* (multidrug and toxic compound extrusion) or *ALMT* (Al-activated malate transporter) families [7–9].

Unlike the external Al exclusion, the internal tolerance mechanisms are achieved by modification of root cell wall and subsequent sequestration of absorbed Al into vacuoles [10]. Plant root cell wall is the first barrier for Al getting into cells, where more than 90% Al is bound [11]. As one of the important components in cell wall, pectin exists in highly methylated form, but can transform into negatively charged demethylation type by pectin methylesterases (PMEs), leading to more Al ions binding [12]. Al sensitive rice genotype exhibited a higher PME activity and demethylated pectin proportion [13]. Furthermore, the lines with *OsPME14* overexpression accumulated more Al in root tip cell wall and showed more sensitivity to Al stress [14]. In addition, an ABC (ATP-binding cassette) transporter complex formed by OsSTAR1 (sensitive to Al rhizotoxicity) and OsSTAR2 proteins is involved specifically in efflux of UDP-glucose, which possibly results in modification of cell wall [15]. To prevent more Al accumulation in cell wall, plasma membrane localized transporter OsNart1 (Nramp aluminum transporter 1) removes Al specifically from root cell wall into cytosol, and then sequesters Al into vacuoles with the help of tonoplast localized ABC transporter OsALS1 (Al-sensitive 1) in rice [16, 17]. Unlike the most plants accumulating Al in roots, some species such as hydrangea and buckwheat alleviate Al damage by translocating Al from roots to shoots in nontoxic forms [18, 19]. In hydrangea sepal tissue, two aquaporin family members HmPALT1 (plasma membrane aluminum transporter 1) and HmVALT (vacuolar aluminum transporter) were identified as Al transporters, which play important role in Al tolerance [20, 21]. In addition to the genes mentioned above, some transcription factors have also been identified in Al-induced pathways. *OsART1* (*Al resistance transcription factor 1*) is quite important in Al tolerance, because it regulates at least 31 genes to detoxify Al, such as *OsSTAR1*, *OsSTAR2*, *OsNrat1* and so on [15, 16, 22].

Barley is considered as one of the most Al-sensitive cereal crops and also shows a wide genotypic difference in Al tolerance, such as Al-sensitive cultivar Golden Promise and Al-tolerant cultivar Dayton [23, 24]. The well-known tolerant mechanism in barley is related to Al exclusion mediated by citrate transporter HvAACT1 [8]. It was reported that 1-kb insertion in upstream of *HvAACT1* coding sequence could greatly enhance its expression level, resulting in more citrate secretion to chelate Al and higher Al tolerance, as reflected by Al-tolerant cultivars such as Dayton [23, 24]. While the 1-kb insertion was not found in the Al-sensitive cultivars, such as Golden Promise, in our previous study, 1-kb insertion was not observed in an Al-tolerant Tibetan wild barley accession XZ29, which did not secret more citrate and other organic acids under Al stress [23, 24]. On the other hand, GWAS analysis detected two novel loci associated with Al tolerance in the Tibetan wild barley, but not in cultivars [25]. Tibetan wild barley, inhabited in Tibet plateau with extremely harsh environment which is considered as one of the centers of cultivated barley domestication, is rich in genetic diversity of abiotic stress tolerance [26]. Therefore, it may be assumed that other mechanisms different from the known Al exclusion in cultivated barley might exist in Tibetan wild barley in Al tolerance.

Recently, more and more researches have been done to study gene regulation mediated by miRNAs, which bind with target mRNAs through complementary base pairing, leading cleavage to target genes [27]. MicroRNAs are a kind of noncoding small RNAs with the length of 20–24 nucleotides, they play critical roles in many aspects of plant development, metabolism and biotic and abiotic stress responses [27, 28]. Among them, the family of miRNA156 is highly conserved and regulates *SPL* (*Squamosa Promoter Binding-Like*) target genes associated with plant architecture and tuber yield in potato, panicle branching, grain quality and some other traits in rice [29–31]. In addition to development processes, some miRNAs are also involved in stress resistance. For example, microRNA390 could be responsive to heavy metals stress, including Cd and Al toxicity [32, 33]. Thus it is interesting to determine whether Al tolerance in Tibetan wild barley is involved in the special miRNAs and the possible difference in the miRNA associated with Al tolerance between the wild and cultivated barley.

To understand the possible roles that miRNAs and their potential target genes play in Al-tolerant regulatory networks, small RNA libraries from roots of Tibetan wild barley XZ29 and cultivated barley Golden Promise exposed to Al stress and control (without Al treatment) were constructed and submitted to deep sequencing by high-throughput sequencing technology. In addition to the miRNAs reported previously, a number of novel miRNAs were validated in these libraries, which greatly enriches barley microRNA data. Furthermore, analysis of miRNAs and their target genes in response to Al stress provide a new insight into understanding of Al-tolerant mechanism in barley, especially Tibetan wild barley.

## Results

### The difference in Al tolerance between Golden Promise and XZ29

**Fig. 1:**
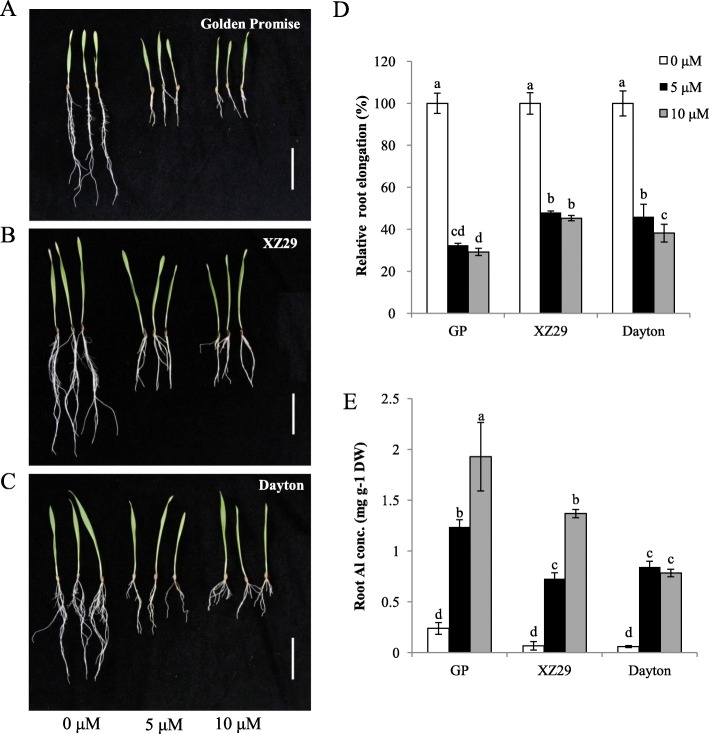
The difference in Al tolerance among three barley genotypes. **a**-**c**, Growth of Golden Promise (GP) (**a**),XZ29 (**b**) and Dayton (**c**) at different Al concentration. Three-day–old seedlings were exposed to Al for 9 days. Relative root elongation (**d**) and the whole root Al concentration (**e**) in three genotypes. Data are means ±SD of six and three biological replicates for **d** and **e** respectively, and means labeled with different letters are significantly different at *p* < 0.05 by Tukey’ test

Under normal condition without Al stress, three genotypes showed much similar root growth and length (Fig. 1a, Additional file 1: Figure S1). After 9 days of Al treatment, the longest root elongation was inhibited more in Golden Promise than in XZ29 and Dayton under the two Al concentrations (5 and 10 μM) (Fig. 1a, b, c, Additional file 1: Figure S1). The relative root elongation was suppressed less in XZ29 than in Dayton at 10 μM Al, although the similar inhibition was found for the two genotypes at 5 μM Al (Fig. 1d).

Root Al concentration increased with external Al level for Golden Promise and XZ29 (Fig. 1d), but for Dayton, there was no obvious difference in root Al concentration between 5 and 10 μM Al treatments. Moreover, XZ29 showed lower root Al concentration than Golden Promise at either 5 μM or 10 μM Al. By contrast, root Al concentration was much higher in XZ29 than in Dayton at 10 μM Al. These results confirmed again that Al-tolerant genotype XZ29 had lower root Al concentration than Al–sensitive cultivar Golden Promise.

### The difference in 1-kb insertion, *HvAACT1* expression level and organic acids secretion between Golden Promise and XZ29

**Fig. 2:**
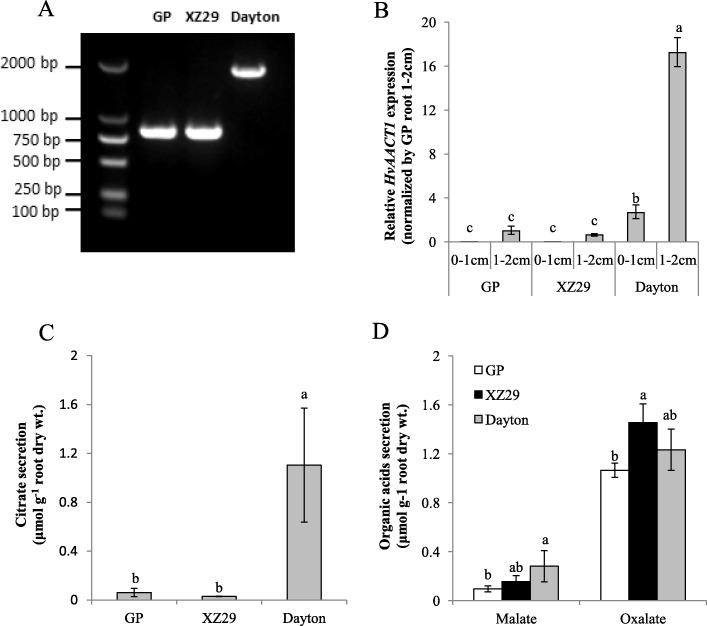
The difference in 1-kb insertion, *HvAACT1* expression and organic acids secretion among three barley genotypes. **a** Detection of 1-kb insertion in the upstream of *HvAACT1* coding region. **b** Expression level of *HvAACT1*. Two root segments (0-1 cm and 1-2 cm from tips) from four-day-old seedlings were sampled after exposure to 10 μM Al for 6 h. *Actin* was used as an internal control and expression relative to 1-2 cm root segments of Golden Promise (GP) is shown. Al-induced citrate (**c**), malate and oxalate (**d**) secretion. Root exudates were collected after four-day-old seedlings were exposed to 10 μM Al for 6 h. Data in **b**, **c** and **d** are means ± SD of three biological replicates and means labeled with different letters are significantly different at *p* < 0.05 by Tukey’ test

To understand whether *HvAACT1* is responsible for the difference of Al tolerance between XZ29 and Golden Promise, 1-kb insertion, *HvAACT1* expression level and organic acids secretion were analyzed. The results showed that both Golden Promise and XZ29 had no 1-kb insertion and much lower *HvAACT1* expression level and less citrate secretion in comparison with Dayton which had 1-kb insertion (Fig. 2a, b, c). In addition, there was no significant difference in malate and oxalate secretion among these three genotypes (Fig. 2d). Obviously citrate secretion, which is associated with 1-kb insertion and high Al tolerance for Dayton, cannot explain the Al stress tolerance for XZ29.

### The difference in small RNA deep sequencing between Golden Promise and XZ29

To identify the miRNAs in response to Al stress, eight small RNA libraries were constructed from roots of Golden Promise and XZ29 in control and Al-treated conditions. Totally 11,574,070, 11,798,931, 11,004,065 and 12,720,633 raw reads were generated by high-throughput sequencing respectively for two genotypes and two treatments (Table 1). After a series data processing, including filtration of small RNAs except miRNAs, 5,487,588, 5,379,474, 5,608,740 and 4,495,143 total valid reads, corresponding to 1,464,860, 2,086,577, 1,850,953 and 1,346,101 unique reads were acquired in the libraries of Golden Promise in control and Al treatment, XZ29 in control and Al treatment, respectively. The majority of valid reads were in length of 19-24 nt, with 24 nt reads being most dominant for Golden Promise after Al treatment, which occupied 27.4% total reads (Additional file 2: Figure S2).

**Table 1:** The profiles of small RNA deep sequencing for the two barley genotypes under different Al treatments

Library type	Golden Promise-Control		Golden Promise-Al		XZ29-control		XZ29-Al	
	Total reads	Unique reads	Total reads	Unique reads	Total reads	Unique reads	Total reads	Unique reads
Raw reads	11,574,070	2,543,454	11,798,931	3,336,429	11,004,065	2,783,930	12,720,633	2,389,395
Cut adapter and length filter	3,207,755	967,414	2,299,195	1,121,113	2,424,530	830,285	2,849,733	908,411
Junk reads	120,196	23,277	113,489	34,527	96,376	25,693	132,744	20,175
Rfam	1,164,361	37,372	1,586,198	41,593	1,177,236	34,644	2,698,268	38,246
mRNA	2,052,214	61,746	3,482,187	65,806	2,162,010	53,694	3,360,733	90,151
Repeats	18,200	713	11,739	342	15,180	543	49,047	848
valid reads	5,487,588	1,464,860	5,379,474	2,086,577	5,608,740	1,850,953	4,495,143	1,346,101

### Identification of the miRNAs expressing in the two barley genotypes under different Al treatments

**Fig. 3:**
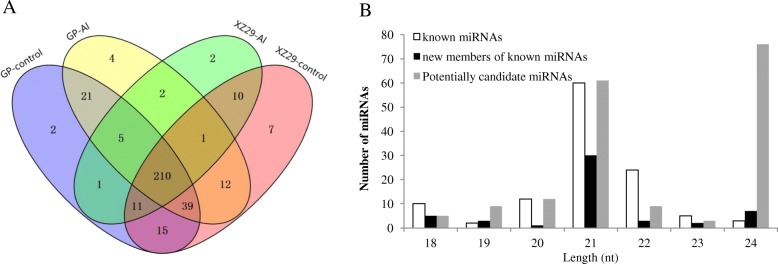
The identified miRNAs from roots of Golden Promise (GP) and XZ29. **a** Number of miRNAs in control and Al-treated roots of two genotypes. **b** Length distribution of total identified miRNAs. A total of 342 miRNAs were found. They were divided into three classes: known miRNAs, new members of known miRNAs and potentially candidate miRNAs

All valid reads from eight small RNA libraries were blasted to miRBase (release 21), which is composed of 35,828 mature miRNAs from 223 species including 72 plant species. A total of 342 miRNAs were identified in the roots of Golden Promise and XZ29, of which 296 miRNAs were shared by the two genotypes (Fig. 3a, Additional file 3: Table S1). All these miRNAs could be divided into three classes, i.e. known miRNAs, new members of known miRNAs and potentially candidate miRNAs, respectively. Known miRNAs included 116 members, perfectly or near perfectly matching to mature miRNAs from barley or other plant species (two mismatches were allowed). New members of known miRNAs included 51 miRNAs, and no mature miRNAs in miRBase were matched to them, but they corresponded to one strand of precursors. There were 175 potentially candidate miRNAs to which no matter mature miRNAs or precursors could match. They were generally at low expression level. Length distribution of these three classes of miRNAs showed that 21 nt sequences were the most abundant in known miRNAs and new members of known miRNAs, while 24 nt sequences were dominant in potentially candidate miRNAs (Fig. 3b).

### Function analysis of miRNA targets in barley

Putative targets for 178 miRNAs were identified while for numerous potentially candidate miRNAs targets were not found (Additional file 3: Table S1). Among 178 miRNAs, 103 miRNAs were predictably associated with 136 target genes, and based on degradome analysis, the rest 75 miRNAs regulated other 136 target genes. Many target genes are transcription factors, indicating showing that miRNAs play important roles in regulatory networks. For example, *SPL* transcription factor 2 and 3 were negatively regulated by ata-miR156a-5p, *ARFs* (*auxin response factors*) was antagonized by ata-miR160a-5p, *NAC* (*NAM, ATAF1/2 and CUC2*) transcription factors were dampened by miR164 family members.

### Identification of miRNAs responsive to Al stress

A total of 50 highly expressed miRNAs in XZ29 and Golden Promise were responsive to Al stress (Table 2). Among them, 17 miRNAs were up-regulated, 24 miRNAs were down-regulated and 8 miRNAs remained unchanged in XZ29. However, 29 miRNAs were responsive to Al in Golden Promise. These miRNAs included known miRNAs such as ata-miR156a-5p and hvu-miR166a, new members of known miRNAs such as hvu-MIR159a-p5 and ata-MIR169d-3p and potentially candidate miRNAs, such as PC-miR1, PC-miR2, PC-miR4 and PC-miR6. Potentially candidate miRNAs PC-miR1 and PC-miR2 were only expressed in XZ29 while PC-miR4 was specifically expressed in Golden Promise. Degradome analysis demonstrated that 24 miRNAs, including ata-miR160a-5p, osa-miR319a-3p.2-3p, ata-miR393-5p, ata-miR396a-5p and ata-miR396e-5p were associated with 46 target genes, including *auxin response factors*, *TCP family transcription factor 4*, *HvAFB*/*HvTIR1* and *growth-regulating factors* (Table 2).

**Table 2:** miRNAs in response to Al stress

miRNA name	GP^a^	XZ29^b^	Transcript	Annotation	Degradome detection^c^
ata-miR156a-3p	0.20	0.89	HORVU3Hr1G072810.1	Gibberellin 2-oxidase	Y
ata-miR156a-5p	−0.49	0.91	HORVU3Hr1G094730.2	Squamosa promoter-binding-like protein 2	Y
			HORVU6Hr1G019700.2	Squamosa promoter-binding-like protein 3	Y
bdi-miR156h-3p	0.33	−1.15	HORVU4Hr1G025850.7	Structure-specific endonuclease subunit slx1	N
			HORVU4Hr1G012480.3	Leucine-rich receptor-like protein kinase family protein	N
hvu-miR159a	−0.21	−0.67	HORVU3Hr1G079490.4	MYB domain protein 33	Y
hvu-MIR159a-5p	−0.44	−1.36	HORVU1Hr1G088510.1	Mitogen-activated protein kinase 16	N
osa-miR319a-3p.2-3p	−0.35	0.76	HORVU5Hr1G103400.1	TCP family transcription factor 4	Y
			HORVU2Hr1G060120.1	TCP family transcription factor 4	Y
ata-miR160a-5p	−0.44	−0.77	HORVU2Hr1G089670.2	Auxin response factor 10	Y
			HORVU7Hr1G101270.6	Auxin response factor 16	Y
			HORVU6Hr1G026750.1	Auxin response factor 18	Y
			HORVU1Hr1G041770.6	Auxin response factor 22	Y
ata-miR164c-3p	0.37	0.63	HORVU6Hr1G088160.5	Quinone oxidoreductase	Y
hvu-miR166a	−0.82	−1.61	HORVU5Hr1G010650.1	Homeobox-leucine zipper protein family	Y
			HORVU5Hr1G061410.29	Homeobox-leucine zipper protein HOX10	Y
			HORVU0Hr1G010250.3	Homeobox-leucine zipper protein HOX32	Y
			HORVU1Hr1G041790.2	Homeobox-leucine zipper protein family	Y
ata-miR166a-3p	−0.54	−1.84	HORVU0Hr1G010250.3	Homeobox-leucine zipper protein HOX32	N
ata-miR166a-5p	−0.71	−0.03	HORVU5Hr1G056820.4	Histidine protein methyltransferase 1 homolog	N
ata-miR166d-5p	−0.53	−0.29	HORVU4Hr1G018020.2	F-box/WD-40 repeat-containing protein	N
ata-miR167a-5p	−0.63	−0.17	HORVU2Hr1G121110.32	Auxin response factor 6	Y
ata-miR167b-3p	−0.92	−0.71	HORVU1Hr1G075520.2	Jacalin-related lectin 3	N
ata-miR167b-5p	0.58	−0.23	HORVU2Hr1G059280.1	SWI/SNF complex subunit SWI3C	N
tae-miR167c-5p	0.64	−2.00	HORVU1Hr1G077630.2	Ubiquitin carboxyl-terminal hydrolase 25	N
			HORVU2Hr1G059280.1	SWI/SNF complex subunit SWI3C	N
ata-miR167f-3p	0.29	0.84	HORVU4Hr1G016990.3	Cysteine desulfurase	N
hvu-miR168-3p	−0.71	1.04	HORVU5Hr1G037570.4	Receptor-like protein kinase	N
hvu-miR168-5p	−0.55	−0.83	HORVU1Hr1G055570.4	WD repeat-containing protein WRAP73	Y
ata-miR169i-5p	0.42	−1.58	HORVU5Hr1G092700.17	Nuclear transcription factor Y subunit A-10	Y
			HORVU4Hr1G075830.4	Nuclear transcription factor Y subunit A-3	Y
			HORVU6Hr1G081080.12	Nuclear transcription factor Y subunit A-5	Y
ata-miR169d-5p	−0.46	−0.86	HORVU5Hr1G092700.17	Nuclear transcription factor Y subunit A-10	Y
			HORVU4Hr1G075830.4	Nuclear transcription factor Y subunit A-3	Y
			HORVU6Hr1G081080.12	Nuclear transcription factor Y subunit A-5	Y
ata-MIR169d-3p	−0.91	−1.82	HORVU5Hr1G089950.4	Chromodomain-helicase-DNA-binding protein Mi-2 homolog	N
ata-miR169h-3p	−0.81	0.18	HORVU1Hr1G075540.3	Mitochondrial processing peptidase alpha subunit	Y
ata-miR171b-5p	−0.70	0.14	HORVU5Hr1G081160.4	U-box domain-containing protein 73	N
ata-miR171a-5p	−1.42	−1.15	HORVU2Hr1G076620.7	T-complex protein 11	Y
ata-miR172b-3p	−0.63	−0.55	HORVU5Hr1G112440.1	Ethylene-responsive transcription factor 10	Y
			HORVU1Hr1G011800.24	AP2-like ethylene-responsive transcription factor	Y
ata-miR390-5p	−1.86	−0.56	HORVU7Hr1G007520.1	Leucine-rich repeat receptor-like protein kinase family protein	N
ata-miR393-5p	−0.16	−1.57	HORVU2Hr1G070800.3	HvAFB	Y
			HORVU1Hr1G021550.4	HvTIR1	Y
ata-miR394-5p	−1.18	−0.30	HORVU1Hr1G043940.3	Protein TIC110, chloroplastic	N
			HORVU6Hr1G018370.1	Calnexin 1	N
ata-miR396a-5p	0.16	−1.20	HORVU7Hr1G008680.14	Growth-regulating factor 5	Y
			HORVU4Hr1G010080.6	Growth-regulating factor 6	Y
			HORVU4Hr1G003440.12	Growth-regulating factor 9	Y
ata-miR396e-5p	0.51	−1.46	HORVU7Hr1G008680.14	Growth-regulating factor 5	Y
			HORVU4Hr1G010080.6	Growth-regulating factor 6	Y
			HORVU4Hr1G003440.12	Growth-regulating factor 9	Y
osa-miR444a-3p.2	−0.14	−0.82	HORVU2Hr1G080490.1	MADS-box transcription factor 27	Y
ppt-miR477h	1.34	2.83	HORVU1Hr1G050450.2	Replication factor C subunit 5	N
osa-miR827	−0.46	−1.26	HORVU6Hr1G065710.13	SPX domain-containing membrane protein	N
ata-miR1432-5p	−1.56	0.21	HORVU1Hr1G094160.1	Calmodulin like 43	Y
			HORVU5Hr1G111520.1	EF hand calcium-binding protein family	Y
hvu-miR5048a	0.36	−1.68	HORVU7Hr1G065130.1	Receptor kinase 2	Y
			HORVU7Hr1G043150.1	Protein kinase superfamily protein	Y
tae-miR7757-5p	0.66	−0.55	HORVU5Hr1G086040.7	NBS-LRR disease resistance protein, putative	Y
tae-MIR9662a-5p	−0.38	2.33	HORVU5Hr1G123930.2	Beta-fructofuranosidase, insoluble isoenzyme 3	N
ata-MIR9863a-5p	0.58	1.39	HORVU5Hr1G007750.22	FAR1-related sequence 3	N
			HORVU1Hr1G004650.4	Purple acid phosphatase 22	N
ppt-miR894	−0.02	2.08	HORVU7Hr1G041460.1	2-oxoglutarate (2OG) and Fe(II)-dependent oxygenase superfamily protein	Y
hvu-miR5051	0.04	−0.92	HORVU7Hr1G054660.6	Chromosome 3B, genomic scaffold, cultivar Chinese Spring	N
			HORVU4Hr1G083260.5	DnaJ homolog subfamily B member 4	N
bdi-miR5054	−1.98	2.07	HORVU4Hr1G003990.3	RNA-binding protein 1	Y
			HORVU6Hr1G088580.4	Zinc finger (C3HC4-type RING finger) family protein	Y
osa-miR5072	−1.21	1.43	HORVU3Hr1G075970.2	Pectate lyase family protein	N
gma-miR6300	0.86	3.08	HORVU4Hr1G052010.2	WRKY DNA-binding protein 46	Y
			HORVU0Hr1G035440.1	Non-specific phospholipase C4	Y
ptc-miR6478	1.04	3.46	HORVU7Hr1G076700.1	Myosin-J heavy chain	N
ath-miR8175	−2.46	1.96	HORVU5Hr1G085710.3	Aquaporin-like superfamily protein, HvNIP1;2	N
PC-miR1	0.00	−0.73	HORVU4Hr1G042240.2	Hexosyltransferase	N
PC-miR2	0.00	1.26	HORVU1Hr1G074900.1	BZIP transcription factor	N
PC-miR4	2.11	0.00	HORVU6Hr1G035300.19	U11/U12 small nuclear ribonucleoprotein 25 kDa protein	N
PC-miR6	−0.69	0.70	HORVU6Hr1G076340.1	Glycosyltransferase family 61 protein	N

^a^GP and ^b^XZ29 represent the fold change between Al treatment and control normalized reads in Golden Promise and XZ29, respectively. It was calculated as the formula: fold change = log_2_ (Al reads / control reads). miRNAs were significantly up-regulated with fold change≥0.5, down-regulated with fold change ≤ − 0.5, unchanged with |fold change| < 0.5. ^c^Degradome detection shows the target genes of miRNAs. Y and N indicate target gene in or not in the degradome sequencing library

### Transcript analysis of miRNA target genes

**Fig. 4:**
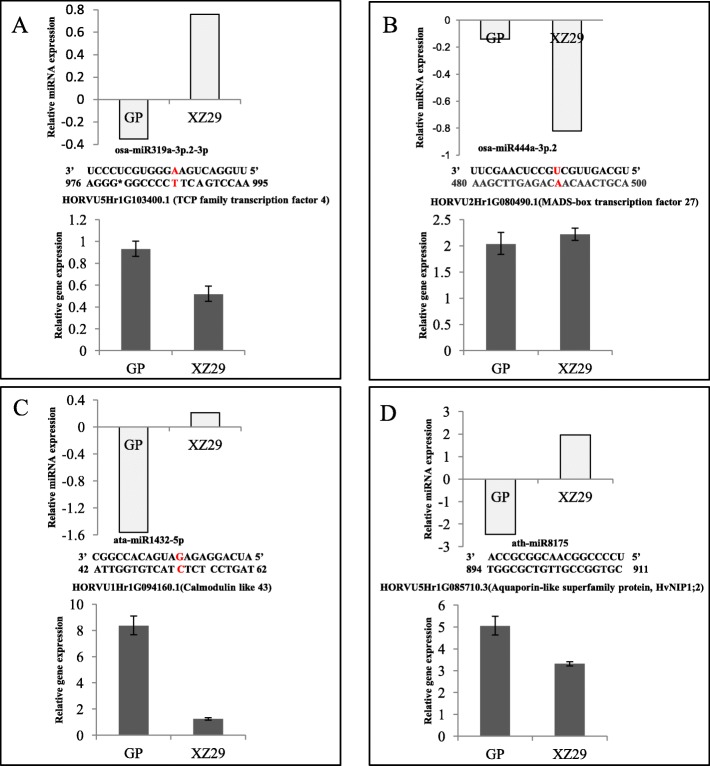
Expression of miRNAs and their targets in Golden Promise (GP) and XZ29, listed as **a** osa-miR319a-3p.2-3p, **b** osa-miR444a-3p.2, **c** ata-miR1432-5p and **d** ath-miR8175. Each panel value of relative miRNA expression represented fold change between Al treatment and control normalized reads from small RNA sequencing. Fold change was calculated as log_2_ (Al reads/control reads). Data in miRNA expression are means of two biological replicates while in gene expression are means of two biological replicates and two technical replicates. Red letter in sequences indicated miRNA cleavage sites on targets with degradome evidence

According to gene annotations, putative targets for 50 miRNAs were associated with a variety of biological processes such as auxin responses, growth regulation and so on (Table 2). Four target genes from Al responsive miRNAs were randomly selected for qRT-PCR analysis to confirm reads of high-throughput sequencing and accuracy of target gene identification. The expression level of osa-miR319a-3p.2-3p was significantly up-regulated and target gene HORVU1Hr1G094160.1 (*TCP4*) was down-regulated in XZ29, however there was no obvious difference for the expression of osa-miR319a-3p.2-3p and its target gene in Golden Promise (Fig. 4a). osa-miR444a-3p.2 was down-regulated in XZ29 and little changed in Golden Promise, while the target gene HORVU2Hr1G08490.1 (*MADS27*) was up regulated by 2.2- and 2.0-fold in XZ29 and Golden Promise, respectively (Fig. 4b). The expression of ata-miR1432-5p was significantly down-regulated and its target gene HORVU1Hr1G094160.1 (*CML43*) was up-regulated in Golden Promise, while XZ29 showed little change in the expression of both ata-miR1432-5p and its target gene (Fig. 4c). As shown in Fig. 4d, ath-miR8175 was up-regulated in XZ29 and down-regulated in Golden Promise. The target gene HORVU5Hr1G085710.3 (*HvNIP1;2*) showed higher expression level in Golden Promise than in XZ29. In short, there was a distinct difference in the expression of some miRNAs as well as their target genes between XZ29 and Golden Promise in responses to Al stress.

## Discussion

Relative root elongation has been widely used for evaluating Al tolerance in plants [15, 23]. In this study, XZ29 was less inhibited in root length and accumulated lower Al in roots than Golden Promise under Al stress, proving that XZ29 was higher Al tolerance than Golden Promise (Fig. 1; Additional file 1: Figure S1). Dayton, a well-known Al-tolerant cultivated barley, showed its high Al tolerance through more citrate secretion mediated by citrate transporter HvAACT1, which was attributed to 1-kb insertion in the upstream of *HvAACT1* coding region [23, 24]. Such a 1-kb insertion was not detected in Al-sensitive cultivar Golden Promise and Al-tolerant Tibetan wild barley accession XZ29 (Fig. 2a). Furthermore, the expression of *HvAACT1* and amount of organic acids including citrate were much lower in XZ29 and Golden Promise than in Dayton (Fig. 2b-d). These results indicated that organic acids efflux from roots into rhizosphere to chelate Al could not account for Al tolerance in XZ29, and some other mechanisms underlying the Al tolerant wild barley should exist.

In the past few years, more and more miRNAs have been identified to be associated with biotic or abiotic stress responses in different plant species [27]. In the current study, a total of 342 miRNAs were found in the two barley genotypes XZ29 and Golden Promise, which greatly enriches the database. Based on the comparison of miRNA expression profiles between XZ29 and Golden Promise in control and Al stress, 50 highly expressed miRNAs responsive to Al stress were identified and their target genes were also detected through degradome analysis and bioinformatics prediction (Table 2).

### Auxin signaling pathway mediated cell wall modification is responsive to Al stress

In addition to the direct inhibition of cell elongation, Al stress also altered ethylene and auxin biosynthesis and accumulation in soybean, affecting root growth [34]. miR393 has been found to be associated with various responses to auxin related stress [35, 36]. Hyposensitivity to auxin was observed in miR393 over-expression lines, resulting in the enhanced sensitivity to salt and drought stresses in rice [35]. But in barley, overexpression of miR393 greatly enhanced Al tolerance through auxin signaling regulation [36]. In the current study, ata-miR393-5p, negatively regulating two auxin receptor genes *HvAFB* (*auxin-signaling F-box*) and *HvTIR1* (*transport inhibitor response*), was significantly down-regulated in XZ29, and little changed in Golden Promise under Al stress. The result was consistent with the report by Bai et al. [36] that the expression of *HvAFB*/*TIR1* was enhanced and their downstream genes *ARFs* in auxin signaling pathway were also promoted. Consequently, ata-miR160a-5p, the negative regulator of *ARF* family members, was suppressed much more in XZ29 under Al stress. Actually it was found that loss-of-function in *arf10*/*16* double *Arabidopsis* mutant showed higher Al tolerance due to different expression of genes encoding proteins involved in cell wall modification, including enhanced PMEIs (pectin methylesterase inhibitors), repressed XTH31 (xyloglucan endotransglucosylase/hydrolase) and XTH7 [37]. Therefore, auxin signaling pathway associated with miR160 and miR393 was associated with Al stress response, which might be a strategy for plants to detoxify Al.

### miR319/TCP4 module regulates Al-induced root growth inhibition

Root length depends on the balance of cell elongation and proliferation. Jasmonic acid and miR396 mediated *GRFs* (*growth-regulating factors*) have been reported to be associated with cell proliferation in *Arabidopsis* [38, 39]. A significantly reduced root length in miR396 overexpression lines was linked with a reduction of dividing cells number in root apical meristem of the model legume *Medicago truncatula*, which showed lower *GRFs* expression [40]. It has been reported that miR319 mediated *TCP4* represses *Arabidopsis* cell proliferation partially through the direct positive regulation of miR396 [41]. On the other hand, miR319/*TCP4* module regulates jasmonic acid biosynthesis, as showed by a recent report, suggesting that overexpression of miR319 in tomato reduced endogenous jasmonic acid level [42]. Recently, it has been revealed that jasmonic acid could enhance Al-induced root inhibition in *Arabidopsis* due to increased microtubule depolymerization, and this progress was regulated by ethylene [43]. In this study, osa-miR319a-3p was up regulated while ata-miR396a-5p and ata-miR396e-5p were down regulated in XZ29, showing the opposite expression patterns. Therefore, the similar regulatory module between miR319/TCP4 and miR396 might be applied in barley to detoxify Al. It can be concluded that miR319 mediated *HvTCP4* was significantly down regulated in XZ29 relative to that in Golden Promise, in order to alleviate Al induced root inhibition aggravated by jasmonic acid. Meanwhile, miR396 expression was also reduced in XZ29, leading to enhanced root cell proliferation.

### Ath-miR8175 regulating HvNIP1; 2 might act as Al transporter

To cope with Al stress, rice has developed a transport system for Al sequestration into vacuoles, which is mediated by OsNart1 and OsALS1 [16, 17]. Members of aquaporin family, which mostly transported non-charged substrates, were involved in Al transport in some plants such as hydrangea [20, 21]. In addition, a recent study reported that plasma membrane localized transporter AtNIP1; 2 was involved in Al uptake from root cell wall into symplasm, and root to shoot translocation in the form of Al-malate complex [44]. Compared with rice and *Arabidopsis*, barley is relatively more Al sensitive, and internal Al detoxification mechanisms including Al transporters are hardly known. In this study, the gene *HvNIP1;2*, negatively regulated by ath-miR8175, was significantly up-regulated in both XZ29 and Golden Promise. However, the expression of ath-miR8175 was greatly increased in XZ29, thus potential upstream genes might differ in XZ29 and lead to enhancement of *HvNIP1; 2*, like the relation between *OsART1* and *OsNart1* [16]. Belonging to nodulin-26 like intrinsic protein (NIP) subfamily of aquaporin family, *HvNIP1; 2* is homologous with *AtNIP1; 2*. Based on these facts, it may be assumed that *HvNIP1; 2* might act as an Al transporter to facilitate Al complex uptake from root cell wall into cytosol, but does not participate in Al translocation from root to shoot.

### Novel miRNAs exclusively expressing in Tibetan wild barley XZ29

In the current study, two novel miRNAs, PC-miR1 and PC-miR2 were exclusively detected in XZ29, and not in Golden Promise. The miRNA PC-miR2 showed higher expression under Al stress in XZ29. This novel miRNA dampened the expression of target gene HORVU1Hr1G074900.1, which encoded BZIP (basic leucine zipper) transcription factor. In common bean, Al stress suppressed the expression of transcription factors such as *BZIP* and *MYB* in drought-induced ABA pathway [45]. However, the relation between Al stress and *BZIP* is hardly known. In addition, the target gene HORVU4Hr1G042240.2 of another novel miRNA PC-miR1, which encoded hexosyltransferase, might be associated with pectin biosynthesis in cell wall. It was well documented that pectin was related to Al tolerance [13]. Higher PME activity in an Al-sensitive rice cultivar was linked with greater proportion of demethylated pectin [13]. Similarly, Al sensitive maize showed low-methylated pectin [46]. Based on these findings, it might be assumed that Golden Promise should have higher cell wall pectin content because of no regulation by miRNAs PC-miR1. More demathylated pectin might be produced due to higher PME activity, resulting in more Al ions binding to cell wall in Golden Promise. As a result, Golden Promise had higher root Al concentration than XZ29 (Fig. 1d). In conclusion, the exclusively expressed novel miRNA PC-miR1 could detoxify Al stress through altering cell wall component, but its precise roles need to be explored in further studies.

In addition to the miRNAs discussed above, some other miRNAs such as osa-miR444a-3p.2 and ata-miR1432-5p could be also involved in Al stress responses. Al stress reduced osa-miR444a-3p accumulation in XZ29 and enhanced the expression of target gene HORVU2Hr1G080490.1 encoding MADS-box transcription factor 27 in both genotypes, with XZ29 being more affected than Golden Promise. It was reported that miR444a overexpression affected rice lateral, primary and adventitious root growth through mediating nitrate signaling pathway [47]. In this study, we found HORVU1Hr1G094160.1, which encoded calmodulin like 43 (CML43) proteins, was greatly up-regulated in Golden Promise, but remained little change in XZ29. However, the physiological roles of most CMLs are still unclear, indicating the potential significance in deciphering their functions.

**Fig. 5:**
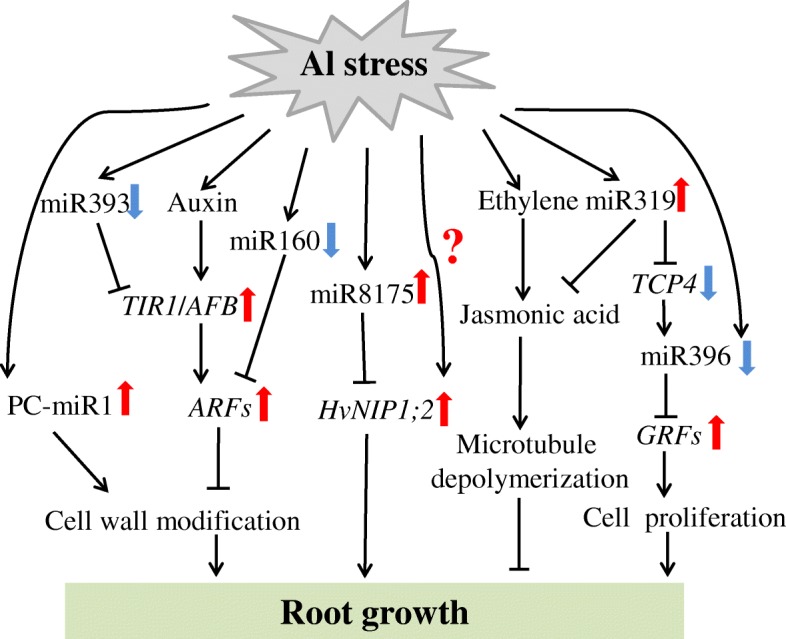
A model presenting Al responses in XZ29. Down regulation of miR160 and miR393, accumulation of jasmonic acid cause root growth inhibition. To alleviate the inhibition, the expression of miR319 and miR396 is significantly changed, resulting in acceleration of cell proliferation. On the other hand, the specifically expressed miRNA PC-miR1 in XZ29 might affect cell wall component through influencing pectin biosynthesis, leading to less Al ions binding to cell wall. Al transporters facilitate Al from cell wall into cytosol and then Al is stored in nontoxic forms

Based on analysis of miRNAs responding to Al stress, we developed a model to reveal Al tolerant strategies in XZ29 (Fig. 5). miR160 and miR393 participating in auxin signaling pathway, PC-miR1 being involved in cell wall pectin biosynthesis and miR319/TCP4 module as well as miR396 taking part in cell proliferation, play important roles in regulation of Al-induced root inhibition. The gene *HvNIP1;2* might be responsible for Al transport. All these findings provide a framework for understanding the roles of miRNAs in the novel mechanism of Al tolerance in the Tibetan wild barley.

## Conclusions

The gene *HvAACT1* and organic acids secretion are not responsible for Al tolerance in the Tibetan wild barley accession XZ29. Small RNA sequencing is an efficient way to detect miRNAs involved in biotic and abiotic stress. In this study, 50 miRNAs were responsive to Al stress in Golden Promise and XZ29. Among them, 17 miRNAs were up-regulated and 24 miRNAs were down-regulated in XZ29. These miRNAs play important roles in Al tolerance in the Tibetan wild barley.

## Methods

### Plant materials and culture conditions

Tibetan wild barley accession XZ29 and a cultivated barley Golden Promise were used to compare Al tolerance, expression level of *HvAACT1* and secretion of organic acids. An Al-tolerant cultivar Dayton was used as a reference genotype. The seeds of XZ29 were obtained from Huazhong Agricultral University, China; and the seeds of cultivars Golden Promise and Dayton were from University of Tasmania, Australia. Barley seeds were disinfected with 3% H_2_O_2_ for 20 min and then soaked in deionized water for 4 h at 22 °C. Finally, seeds were germinated hydroponically on surface of 1.1-L plastic pots filled with aerated 0.5 mM CaCl_2_ solution and placed in a growth chamber (22/18 °C, day/night) at dark environment, then two days later supplied light with 250 μmol m^− 2^ s^− 1^.

XZ29 and Golden Promise were used to generate miRNA data. Seeds of two genotypes were germinated on moist filter papers in a growth chamber under dark environment. After 7 days germination, seedlings were transferred into 5-L plastic containers with aerated one-fifth Hoagland solution and renewed every 3 days.

### Measurement of root elongation and Al concentration

Three-day-old seedlings prepared as described above were exposed to 1 mM aerated CaCl_2_ solution containing 0, 5 and 10 μM Al at pH 4.5. The solution was renewed daily. After treatment for 9 days, root elongation was measured as the length of the longest root. The relative root elongation was calculated as the formula: the longest root length with Al treatment/the longest root length without Al treatment × 100%. The whole roots were harvested and washed three times with 5 mM CaCl_2_ solution. Samples were dried in an oven at 70 °C for 2 days and then digested completely in HNO_3_ solution using a microwave digestion instrument (Multiwave 3000, Anton Paar GmbH, Australia). The concentration of Al in the digested solution was determined by inductively coupled plasma optical emission spectrometer (ICP-OES) (iCAP 6000 series, Thermo Fisher scientific, USA).

### Identification of the 1-kb insertion in the upstream region of *HvAACT1*

DNA was extracted from shoots according to the instructions of MiniBEST Universal Genomic DNA Extraction Kit Ver.5.0 (TaKaRa, Japan). To examine the presence of the insertion in three barley genotypes, upstream fragments of the *HvAACT1* encoding region were amplified by PCR using KOD-FX (Toyobo, Japan) from the genomic DNA. PCR primers (5’-GGTCCAACACTCTACCCTTCCTT-3’and5’-GGTGCGAGTTGCCCCTAGCTATTACAGA-3′) were used as showed by Fujii et al. [23]. The PCR products were separated by electrophoresis of 120 V running for 30 min on a 1% agarose gel using 1 × TAE buffer and stained with 4S green plus nucleic acid stain (Sangon Biotech, Shanghai, China).

### Determination of *HvAACT1* expression

To compare the *HvAACT1* expression level in XZ29, Golden Promise and Dayton, four-day-old seedlings were exposed to 1 mM aerated CaCl_2_ solution containing 5 μM Al at pH 4.5 for 6 h. Two root segments (0–1 cm and 1–2 cm from tips) were sampled and frozen immediately in liquid nitrogen, then stored at − 80 °C before use. RNA was extracted by MiniBEST Universal RNA Extraction Kit (TaKaRa, Japan) according to instructions from manufacture. First strand cDNA was synthesized using Reverse Transcriptase Kit (TaKaRa, Japan). The qRT-PCR reaction consisting of SYBR Green Supermix (Bio-Rad, America) was conducted on real-time PCR System (LightCycler 480®II,96, Roche, Switzerland). Primers of *HvAACT1* (5’-GTTCGCCAAGAACGATCACA-3′ and 5’-AGAGACCAAGCACCACCGTC-3′) were taken from fujii et al. [23]. Actin was used as an internal control by the ΔΔCt method and primers used for *Actin* were 5’-GACTCTGGTGATGGTGTCAGC-3′ and 5’-GGCTGGAAGAGGACCTCAGG-3′ taken from Furukawa et al. [8]. Expression data of *HvAACT1* were normalized with those in the 1–2 cm root tips of Golden Promise.

### Measurement of organic acids secretion

Root exudates were collected from four-day-old seedlings of three barley genotypes exposed to 1 mM aerated CaCl_2_ solution containing 10 μM Al at pH 4.5 for 6 h. Obtained root exudates were passed through 5 g cation exchange resin (Amerlite IR-120H, Sigma) followed by 2 g anion exchange resin (Dowex 1 × 8 resin, 100–200 mesh, chloride form, Sigma). Organic acids retained in anion exchange resin were eluted immediately with 1 M HCl and then the eluent was dried to powder using rotary evaporator at 40 °C. The powder was dissolved in 2 ml of distilled water and passed through a 0.2 μm syringe filter. The concentration of organic acids was determined by ion chromotography (ICS 2000; Dionex).

### Small RNA and degradome library construction and sequencing

XZ29 and Golden Promise were used for small RNA and degradome sequencing. After grown in one-fifth Hoagland solution for 12 days, these two genotypes were treated with 1 mM aerated CaCl_2_ solution containing 0 or 10 μM Al at pH 4.5 for 24 h. A total of eight root samples (two genotypes in control and Al-treated conditions with two replications) each consisting of three plants were harvested. In addition to construction of eight small RNA libraries, all remained samples were mixed well for RNA collection to establish one degradome library. Total RNA was extracted using Trizol reagent (Invitrogen, CA, USA). Approximately 1 μg of total RNA was used to prepare small RNA library following the manufacture of TruSeq Small RNA Sample Prep Kits (Illumina San Diego, USA), while approximately 20 μg of total RNA was used for degradome library construction according to the protocols described previously [48]. Then single-end sequencing (50 bp) were performed on an Illumina Hiseq2500 (Illumina, San Diego, USA) following protocols of the producer. The detailed information about sequencing quality was showed in Additional file 4: Table S2.

### Prediction of miRNA and their targets

The raw reads for small RNA sequencing were processed with the program ACGT101-miR (LC Sciences, USA) to remove adapters, junk reads, low-complexity sequences, mRNA (ftp://ftp.ensemblgenomes.org/pub/plants/release-36/fasta/hordeum_vulgare/cds/Hordeum_vulgare.Hv_IBSC_PGSB_v2.cds.all.fa.gz), repeats (V18.02; http://www.girinst.org/repbase) and common non-coding RNA families including rRNA, tRNA, snRNA and snoRNA (version 11; http://rfam.janelia.org). Subsequently, the remaining clean unique sequences with the length of 18–25 nucleotides were mapped to miRBase (version 21; ftp://mirbase.org/pub/mirbase/CURRENT/). Length variation at both 3’ and 5’ ends and two mismatches inside of the sequence were allowed in the alignment. The unique sequences mapping to barley and other plant mature miRNAs in miRBase were identified as the known miRNAs. The remained unique sequences mapping to the arm of the known precursor hairpin were considered to be new members of known miRNAs. The mapped pre-miRNAs were further compared to barley genome (http://plants.ensembl.org/Hordeumvulgare/Info/Index) to determine genomic locations. Finally, the unmapped sequences were blasted against barley genome, and then the flank 120 nt sequences were extracted to predict secondary structures using RNAfold software (http://rna.tbi.univie.ac.at/cgi-bin/RNAfold.cgi). The criteria were adjusted as follows: (1) number of nucleotides in one bulge in stem is ≤12, (2) number of base pairs in the stem region of the predicted hairpin is ≥16, (3) the free energy for miRNA precursor should be ≤ − 15 kCal/mol, (4) length of hairpin is larger than 50 but less than 200 nt, (5) number of nucleotides in one bulge in mature region is≤4, (6) number of biased errors in one bulge in mature region should be≤2 (7) number of biased bulges in mature region is ≤2, (8) number of errors in mature region is ≤4, (9) number of base pairs in the mature region of the predicted hairpin is ≥12, (10) percent of mature in stem is ≥80. All remaining sequences meeting these parameters were considered to be potential candidate miRNAs (PC-miRNAs). The raw reads for miRNAs were normalized by the global normalization procedures [49].

The program TargetFinder was used to identify miRNA binding sites in terms of bioinformatics analysis. In addition, degradome library was constructed to further predict the target genes of miRNAs. Raw sequencing reads were filtered by Illumina’s Pipeline software (version 1.5) and then CleaveLand3.0 was used for sequencing data analysis. Finally, degradome reads were mapped to barley mRNA database. All function annotation of target genes was taken from barley CDS database in IPK (http://webblast.ipk-gatersleben.de/barley_ibsc/downloads/160517_Hv_IBSC_PGSB_r1_CDS_HighConf_REPR_annotation.fasta.gz).

### Identification of miRNAs responsive to Al stress

miRNAs responsive to Al stress should meet the rules as showed below. (1) One of normalized reads was larger than 100 from two genotypes in Al-treated or control conditions. (2) MFEI (minimal folding free energy index) of newly found miRNAs was larger than 0.85. (3) The fold change between Al treatment and control normalized reads in Golden Promise and XZ29 was calculated as the formula: fold change = log2 (Al reads/control reads). miRNAs were significantly up-regulated with fold change≥0.5, down-regulated with fold change≤ − 0.5, unchanged with |fold change| < 0.5.

### Transcript analysis of miRNA target genes

To validate the small RNA sequencing data, four randomly selected target genes from miRNAs responsive to Al stress were used to perform qRT-PCR analysis. RNA samples used for cDNA synthesis were the same as those for small RNA library construction. All the next procedures were followed as those described in determination of *HvAACT1* expression. The primers of HORVU2Hr1G080490.1 (5’-TCATCGGCAGTTGATGGGAC-3′ and 5’-GGTGGACAAGACTCCCCTTG-3′), HORVU1Hr1G094160.1 (5’-GGGTGTGAAGGACTTGGTGT-3′ and 5’-CTCATCGAAGGCACGGATCA-3′), HORVU5Hr1G103400.1 (5’-GCTCAAGCCGGAGACTACAG-3′ and 5’-CTGCTCGTACAGGAAGGGAG-3′) and HORVU5Hr1G085710.3 (5’-CCCAATTAGTGGCGCTGTTG-3′ and 5’-AGCTCATCCTTGCACTTCGT-3′) were used and *Actin* was taken as an internal control by the ΔΔCt method. Every sample was carried out with two technical replicates.

## Additional files

Additional file1: **Figure S1.** The difference in root elongation of three genotypes under Al stress. Three-day-old seedlings were exposed to Al for 9 days. The root elongation were measured. Data are means +SD of six biological replicates and means labeled with different letters are significantly different at *p* < 0.05 by Tukey’ test. (PDF 225 kb)

Additional file2: **Figure S2.** Length distribution of small RNAs in control and Al-treated roots of Golden Promise and XZ29. (PDF 225 kb)

Additional file3: **Table S1.** Detailed information of total detected miRNAs in Golden Promise and XZ29. (XLSX 60 kb)

Additional file4: **Table S2.** Detailed information of miRNA libraries sequencing. (XLSX 9 kb)

## Abbreviations

ABC: ATP-binding cassette
*AFB*: *auxin-signaling F-box*
ALMT: Al-activated malate transporter
ALS1: Al-sensitive 1
*ARFs*: *auxin response factors*
*ART1*: *Al resistance transcription factor 1*
BZIP: basic leucine zipper
CML43: calmodulin like 43
GWAS: Genome-wide association study
HvAACT1: Al-activated citrate transporter 1
MATE: multidrug and toxic compound extrusion
MFEI: minimal folding free energy index
miRNAs: microRNAs
*NAC*: *NAM, ATAF1/2 and CUC2*
Nart1: Nramp aluminum transporter 1
NIP: nodulin-26 like intrinsic protein
PALT1: plasma membrane aluminum transporter 1
PMEIs: pectin methylesterase inhibitors
PMEs: pectin methylesterases
*SPL*: *Squamosa Promoter Binding-Like*
STAR1: sensitive to Al rhizotoxicity1
*TIR1*: *transport inhibitor response*
VALT: vacuolar aluminum transporter
XTH3: xyloglucan endotransglucosylase/hydrolase
