# Supplementary material for: Identification of microRNAs in response to aluminum stress in the roots of Tibetan wild barley and cultivated barley
Source: BMC Genomics. 2018 Jul 31;19:560. doi: 10.1186/s12864-018-4953-x (PMC6069884; doi:10.1186/s12864-018-4953-x)
Supplement: Supplementary file 1 — Figure S1. The difference in root elongation of three genotypes under Al stress. Three-day-old seedlings were exposed to Al for 9 days. The root elongation were measured. Data are means +SD of six biological replicates and means labeled with different letters are significantly different at p < 0.05 by Tukey’ test. (PDF 225 kb) [file 12864_2018_4953_MOESM1_ESM.pdf]

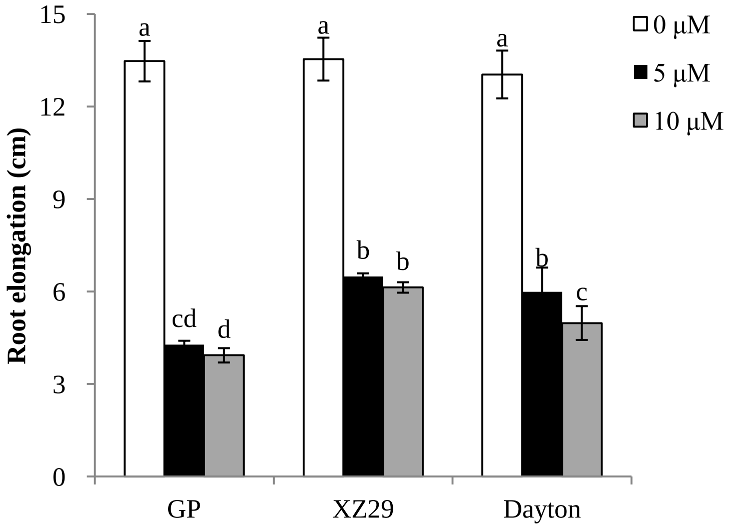

Additional file1: Figure S1. The difference in root elongation of three genotypes under Al stress.

Three-day-old seedlings were exposed to Al for 9 days. The root elongation were measured. Data are means ±SD of six biological replicates and means labeled with different letters are significantly different at $p<0.05$ by Tukey' test.
