# Supplementary material for: Identification of microRNAs in response to aluminum stress in the roots of Tibetan wild barley and cultivated barley
Source: BMC Genomics. 2018 Jul 31;19:560. doi: 10.1186/s12864-018-4953-x (PMC6069884; doi:10.1186/s12864-018-4953-x)
Supplement: Supplementary file 2 — Figure S2. Length distribution of small RNAs in control and Al-treated roots of Golden Promise and XZ29. (PDF 225 kb) [file 12864_2018_4953_MOESM2_ESM.pdf]

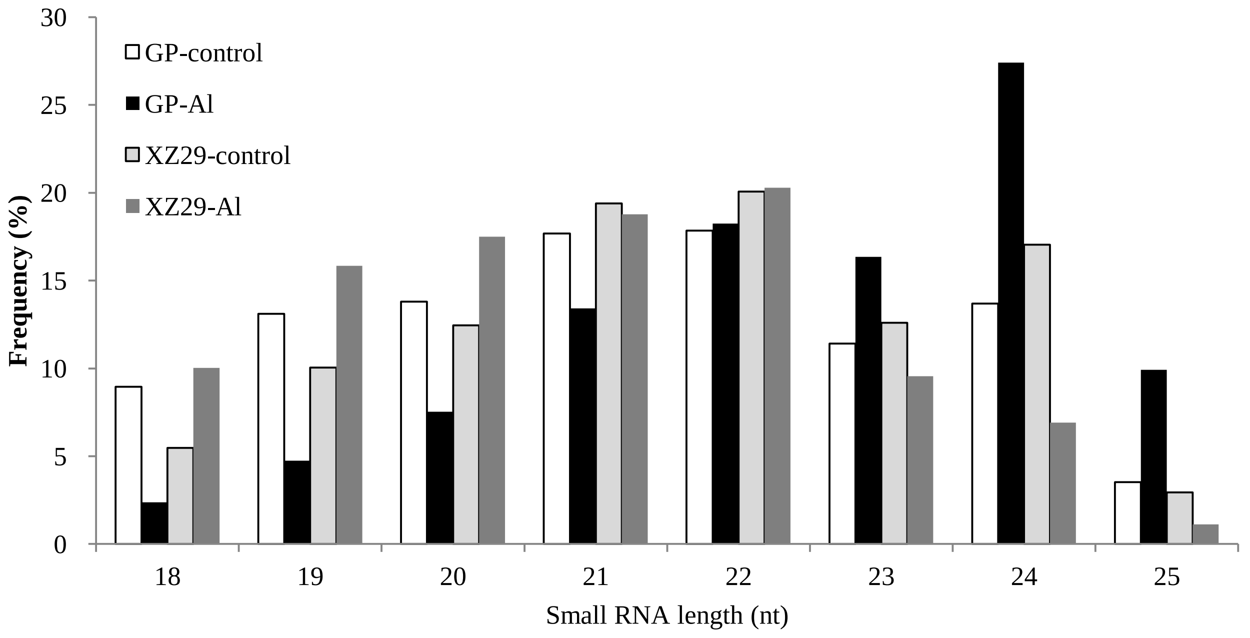

Additional file2: Figure S2. Length distribution of small RNAs in control and Al-treated roots of Golden Promise and XZ29.
